# Nanoformulated Bumetanide Ameliorates Social Deficiency in BTBR Mice Model of Autism Spectrum Disorder

**DOI:** 10.3389/fimmu.2022.870577

**Published:** 2022-05-23

**Authors:** Hui Lv, Xiao Gu, Xingyue Shan, Tailin Zhu, Bingke Ma, Hao-Tian Zhang, Victorio Bambini-Junior, Tiantian Zhang, Wei-Guang Li, Xiaoling Gao, Fei Li

**Affiliations:** ^1^Department of Developmental and Behavioral Pediatric & Child Primary Care, Brain and Behavioural Research Unit of Shanghai Institute for Pediatric Research and Ministry of Education-Shanghai Key Laboratory for Children's Environmental Health, Xinhua Hospital, Shanghai Jiao Tong University School of Medicine, Shanghai, China; ^2^Department of Pharmacology and Chemical Biology, Shanghai Universities Collaborative Innovation Center for Translational Medicine, Shanghai Jiao Tong University School of Medicine, Shanghai, China; ^3^Shanghai Key Laboratory of Brain Functional Genomics (Ministry of Education), School of Life Sciences, East China Normal University, Shanghai, China; ^4^Brain and Behavioral Research Unit of Shanghai Institute for Pediatric Research and Ministry of Education (MOE)-Shanghai Key Laboratory for Children’s Environmental Health, Xinhua Hospital, Shanghai Jiao Tong University School of Medicine, Shanghai, China; ^5^Division of Biomedical and Life Sciences, Faculty of Health and Medicine, Lancaster University, Lancaster, United Kingdom; ^6^Department of Rehabilitation Medicine, Huashan Hospital, Institute for Translational Brain Research, State Key Laboratory of Medical Neurobiology and Ministry of Education Frontiers Center for Brain Science, Fudan University, Shanghai, China

**Keywords:** autism spectrum disorder, bumetanide, nanoparticle, social behavior, microglia

## Abstract

Autism spectrum disorder (ASD) is a prevalent neurodevelopmental disorder with few medication options. Bumetanide, an FDA-approved diuretic, has been proposed as a viable candidate to treat core symptoms of ASD, however, neither the brain region related to its effect nor the cell-specific mechanism(s) is clear. The availability of nanoparticles provides a viable way to identify pharmacological mechanisms for use in ASD. Here, we found that treatment with bumetanide, in a systemic and medial prefrontal cortex (mPFC) region-specific way, attenuated social deficits in BTBR mice. Furthermore, using poly (ethylene glycol)-poly(l-lactide) (PEG-PLA) nanoparticles [NP(bumetanide)], we showed that the administration of NP(bumetanide) in a mPFC region-specific way also alleviated the social deficits of BTBR mice. Mechanistically, the behavioral effect of NP(bumetanide) was dependent on selective microglia-specific targeting in the mPFC. Pharmacological depletion of microglia significantly reduced the effect of nanoencapsulation and depletion of microglia alone did not improve the social deficits in BTBR mice. These findings suggest the potential therapeutic capabilities of nanotechnology for ASD, as well as the relevant link between bumetanide and immune cells.

## Highlights

1. Systemic or mPFC-specific delivery of bumetanide improves social deficit in BTBR mice
2. Nanoformulated bumetanide with microglia-specific targeting capability in mPFC improves social deficit in BTBR mice
3. Microglia depletion in the mPFC fails to affect social deficiency in BTBR mice
4. The therapeutic effects of nanoformulated bumetanide depend on the presence of mPFC microglia

## Introduction

Autism spectrum disorder (ASD) is a highly prevalent neurodevelopmental disorder characterized by social interaction and communication deficits and repetitive patterns of interests, with no efficient, FDA-approved pharmacological options available for its core symptoms (1, 2). Bumetanide, an FDA-approved loop diuretic, has been proposed as a viable candidate to treat core symptoms of ASD. To elucidate the efficacy and molecular mechanisms of bumetanide, rodent models with phenotypes relevant to the core feature of ASD have been widely used (3). BTBR T+tf/J (BTBR) mice were derived from the inbred strain carrying the at (nonagouti; black and tan), T (brachury), and the Itpr3tf (tufted) mutations with marked ASD-like behavior phenotypes such as social deficits and repetitive behaviors (4, 5). Indeed, the ranges of ASD-like phenotypes in BTBR mice highlight this model as a useful preclinical tool for evaluating therapeutic strategies.

One of the mechanisms involved in the manifestation of ASD phenotype is an imbalance of excitatory and inhibitory neurotransmission, which suggests a potential therapeutic target for drug discovery (6). Recent evidence from both preclinical and clinical studies has implicated bumetanide as a promising candidate for ASD therapy (7–10), yet the efficacy of bumetanide remains unclear (11, 12). The classical mechanism explains the action of bumetanide in the regulation of neuronal intracellular chloride flux by inhibition of the sodium (Na^+^)-potassium(K^+^)-Cl^-^transporter isoform1 (NKCC1, *SLC12A2*) and the restoration of GABA polarity in the brain (7). Maternal bumetanide treatment could shift GABA from excitation to inhibition state while rescuing autistic-like behaviors in the offspring of animal models of ASD (7, 8). Clinical evidence has shown that bumetanide could improve the symptoms including social communication and restricted interest of ASD patients (13). This action of bumetanide is mediated by GABA since the decreased insular GABA was observed in ASD patients with bumetanide (10, 14). Despite the effects of bumetanide that have been demonstrated in animal models and patients with ASD, the effects of bumetanide have remained controversial, mainly because the brain region and cell-type specific mechanisms(s) underlying bumetanide in ASD are unclear.

The medial prefrontal cortex (mPFC) is a brain region mechanistically linked to multiple cognitive functions, including social behaviors (15–17). In mice, excitation of excitatory neurons *via* optogenetic manipulation in the mPFC leads to social deficits (15), whilst lesion of the mPFC reduces social behaviors (16). In addition, functional magnetic resonance imaging (fMRI) (18) and functional near-infrared spectroscopy (fNIRS) (19) have revealed the involvement of mPFC in social-associated tasks in humans, and dysfunctional mPFC activity in patients with ASD (20). Considering the critical involvement of mPFC in social behaviors, we thus tried to investigate whether the mPFC acts as a critical brain region underlying the therapeutic actions of bumetanide in ASD.

Despite the majority of studies have examined the neuronal mechanism that underlies bumetanide’s action, the involvement of other non-neuronal mechanisms cannot be completely excluded. Bumetanide is a non-selective NKCC1 inhibitor. Notably, NKCC1 expression is found in several non-neuronal cell types of the brain, including microglia (21). Furthermore, a growing body of evidence suggests that microglia, the resident immune cells in the brain, have a central role in the pathophysiology of ASD (22–26). Microglia dysfunction in ASD has been identified by autopsy and positron emission tomography studies, which showed increased numbers and altered morphology of microglia cells (22, 23), and genomic transcriptional analysis revealed an altered expression of microglia-specific genes in cortical regions of patients with ASD (24, 25). In addition, analysis with positron-emission tomography (PET) has revealed increased microglial activation in young adult patients with ASD (26). As the cell-specific effects of bumetanide have not been extensively characterized (due to its non-selective action), and taking into consideration that none of the previous studies have selectively targeted microglia (despite the evidence demonstrating its important role in ASD) using bumetanide in ASD, we hypothesized that microglia can be one of the primary targets of bumetanide. In previous studies, nanoparticles were used to induce a microglia-specific accumulation of molecules in the brain; this approach can help overcome the constraint of targeting microglial cells specifically (27). The development of a nano-delivery system has provided a new framework for targeted delivery and release for different diseases. For instance, a nano-delivery system established a promising therapeutic approach in Alzheimer’s disease (AD) (28).

In this study, we developed a strategy to evaluate the effects of bumetanide encapsulated in a core of polyethylene glycol-polylactic acid (PEG-PLA) nanoparticles [NP (bumetanide)], using the BTBR mice model of ASD. BTBR mice displayed autistic-like behavioral including social impairments and excess of repetitive behaviors and characterized by aberrant inflammatory phenotypes in both peripheral and central nervous immune system which provided a unique model of ASD to investigate the possible link between bumetanide and immunity. Initially, to characterize the efficiency of the nanoparticles and the specific brain region underlying bumetanide’s mechanism, we tested behavior changes in BTBR mice induced by systemic and mPFC region-specific delivery of bumetanide. Furthermore, we sought to identify the microglia-specific mechanisms involved in the therapeutic effects of bumetanide. For this purpose, we used NP(bumetanide) to target microglia in mPFC. We examined the therapeutic effect of NP(bumetanide) in BTBR mice, as well as how pharmacologically depletion of microglia affects social deficit and the therapeutic effect of NP(bumetanide).

## Materials And Methods

Male C57BL/6 mice were obtained from Shanghai Jihui Laboratory Animal Co., Ltd (Shanghai, China). BTBR mice were obtained from Shanghai model organisms center, Inc. (Shanghai, China). Mice were housed in a standardized environment with a 12 h light/dark cycle and free access to food and water. Behavioral experiments were performed during the light phase of the cycle and mice were randomly grouped. All experimental protocols were approved by the Animal Ethics Committee of East China Normal University (Shanghai, China) and all efforts were made to minimize animal suffering and reduce the number of mice used. All mice used in this experiment were males.

### Drug and Drug Administration

The bumetanide was purchased from Sigma (catalog no. 28395-03-01, USA). For intraperitoneal administration, bumetanide was first dissolved in DMSO and then diluted in 3% Tween80-NS solution to final concentration. Intraperitoneal administration of bumetanide (i.p., 10 mg/kg) was performed daily for 7 days in BTBR mice (6 weeks). For mPFC region-specific administration, bumetanide (100 μM) was dissolved in DMSO and then diluted in NS solution to final concentration. mPFC administration was performed daily for 3 days in BTBR mice (6 weeks). The dose of bumetanide was selected based on previous laboratory results and paper (29, 30).

### Compounds

PLX3397 was purchased from SelleckChem (S7818, China) and formulated in AIN-76A standard chow by Shanghai biopikeChem at 290 mg/kg doses.

### Nanoparticles Preparation

Briefly, NP(bumetanide) was prepared as described previously (27, 31). To address the low solubility of bumetanide during fabrication, we added methyl bonds to bumetanide to promote dissolution and facilitate fabrication into nanoparticles. The previous finding has defined that the bumetanide ester could release bumetanide *in vivo* upon hydrolysis. Firstly, 10 mg of MePEG-PLA and 0.5 mg of bumetanide prodrugs were dissolved in 1 ml of dichloromethane. Afterward, 2 ml of aqueous sodium cholate (1%, w/v) was added to the solution and then sonicated on ice (220 w, 2 min) using a probe sonicator (Ningbo Scientific Instruments Co., Ltd., China) to form O/W emulsions. The emulsion was diluted into an 18 ml of sodium cholate solution (0.5%, w/v) with magnetic stirring for 5 min and evaporation of dichloromethane with a ZX-98 rotary evaporator (Shanghai Institute of Organic Chemistry, China). The nanoparticles were collected by centrifugation at 14,000 × g for 45 min and separated by a 1.5 × 20 cm sepharose CL-4B column. The fluorescently labeled nanoparticles were prepared by a process similar to that of NP(bumetanide), using rhodamine as the fluorescent probe. Rhodamine (0.2mg) was dissolved by dichloromethane in 1 ml of dichloromethane, and then the procedure for the preparation of NP (bumetanide) was followed.

### Surgical Procedures and Micro-Infusion of Bumetanide Administration

For cannula implantation, 5 weeks BTBR mice were anesthetized with 5% chloral hydrate (5 mg/kg) and gently placed in a stereotaxic frame (RWD Life Science, China). Cannulae (62203, RWD Life Science, China) were bilaterally impanated at 1.0 mm above. Stereotaxic coordinates according to the Paxinos and Watson mouse brain atlas (32). The target area to the mPFC at the following coordinates: +1.70 mm; posterior to bregma, ± 1.65 mm; lateral to the midline, and -1.05 mm; dorsoventral. The cannula was angled at 30°, positioned with acrylic dental cement, and secured with cranial screws. A stylet was placed in the introducer cannula to prevent occlusion. Mice were allowed to recover from surgery for several days prior to experimental manipulation. For drug administration, the infusion tube was connected to a microinjector driven by a microinfusion pump (KDS 310, KD Scientific, USA) through PE20 tubing. 0.5 ul per side of bumetanide was infused into the mPFC at a rate of 0.5 ul/min. The infusion tube was left in place for an additional 3 min to allow the diffusion of medicine.

### Behavior Protocol

BTBR mice were examined after 7 days (i.p.) or three times (mPFC infusion) of treatment with bumetanide or vehicle. On the testing day, mice were transferred into the testing room for accommodation for 1 h. During the day of behavioral testing, mice were treated with bumetanide, and tests began 30 min after injection. The devices were cleaned with 70% ethanol after trails to avoid odor cue.

*Three-Chamber test.* The three-chamber test consisted of three stages in one session to assess different social aspects of the mice. The test used a Plexiglas apparatus (60 × 40 × 25 cm) containing three chambers with an empty wire cage (8 × 12 cm) in the side chambers. ANY-maze tracking system software (Stoelting Co, USA) with a camera was used to track the position of the mice during each tracking session. Briefly, in the habituation stage, test mice were placed in the central chamber only for 5 min and then were allowed to explore the whole apparatus with its empty wire cage for 10 minutes. In the sociability stage, a stimulus mouse (stranger 1, S1, same age and sex) was randomly placed in one of the empty wire cages and the test mice were brought back to explore the apparatus for 10 min. After the social ability stage, a new stimulus mouse (stranger 2, S2, same age and sex) was inserted into the previous empty wire cage and the subject mice were allowed to explore the chambers for another 10 min. The time spent by the animals in each chamber was analyzed by ANY-maze tracking system software. Mice with a preference for one side of the chamber were excluded from the data analysis.

*Open field test.* The open field test was used to assess the effects of locomotor responses to novel environments in mice. We conducted the open field test in a square Plexiglas apparatus (40 × 40 × 35 cm) under diffuse light. During the experiment, mice were gently placed in the center square of the apparatus and allowed to explore freely for 30 min. At the end of each trial, the apparatus was cleaned and the animals were returned to cages. Tru scan (Coulbourn Instruments, USA) activity software was used to detect the activity of the mice and record data. The total distance traveled during the 30 min was analyzed.

*Elevated plus maze*. The elevated plus maze could be used to assess anxiety-like behaviors in rodents. This apparatus consists of two walled elevated arms and two open arms that branch off a central platform forming a plus shape. The apparatus was raised to a height of 50 cm above the ground. For testing, subject mice were placed in the center area facing the close arm and allowed to explore for 5 min. ANY-maze tracking system software was used to track the position of the mice during the session and the time spent in the open arms of the maze was analyzed.

*Elevated zero maze*. The elevated zero maze was used to assess anxiety-like behaviors in rodents. The apparatus consists of two open (stressful) and two enclosed (protecting) elevated arms that form a zero or circle elevating it 50 cm above the ground. Quadrant lanes of the apparatus were 5 cm wide. The subject mice were placed randomly in the boundary between a close and an open arm, facing the close arm and allowed for exploring 5 min. ANY-maze tracking system software was used to track the position of the mice during the session and the time spent in the open arm of the maze was analyzed by ANY-maze tracking system software.

*Grooming test.* The grooming test was used to examine the repetitive behaviors of the mice. The test cage was lined with bedding (<1cm) in order to reduce neophobia but prevent digging. For the self-grooming test, mice were placed individually in a new clean cage for 15 min habituation and recorded for 10 min using Mouse Home Cage Behavior Analyzing System (Clever Sys. Inc, USA). The time spent self-grooming was analyzed by Mouse Home Cage Behavior Analyzing System.

### Immunohistochemistry

Briefly, mice were deeply anesthetized with 5% chloral hydrate and transcranial perfused with 40-60 ml of 1 × PBS followed by 20 ml 4% paraformaldehyde. The brain was removed from the skull and immersed into 0.1M PBS containing 4% paraformaldehyde and incubated at 4°C overnight. Coronal brain sections of 45-μm thickness (Leica VT1200S, Germany) were made with oscillating slices. The section was pasted on the poly Lys-coated glass slide and baked at 60°C for 50 minutes. Sections were washed 5 minutes with 1 × PBS for one time. The section was incubated in a blocking buffer composed of PBS containing 0.3% Triton X-100 and 5% normal goat serum for 90 min at room temperature. The sections were then incubated with the primary antibody in the blocking buffer at 4°C for 24 h. The primary antibodies used in this study were rabbit polyclonal anti-Iba1 antibody (1:1000 dilution, 019-19741, wako, Japan), anti-NeuN antibody (1:500, ab104224, Abcam) and anti-GFAP antibody (1:500, 60190-1, proteintech, USA). After incubation, the sections were washed for 5 min with 1 × PBS containing 0.1% Tween-20 for 3 times. For immunofluorescent staining, the sections were incubated with Alexa Fluor 488-labeled secondary antibodies (1:1000 dilution; Life Technologies, USA) for 2h at RT. The sections were washed 5 minutes for three times with PBS containing 0.1% Tween-20. Slides were mounted in the dark with glass coverslips using mounting media containing 4’,6-Diamidina-2-phenylindole (P0131, Beyotime, China). The signal of slices was captured by immunofluorescence microscopy (Leica DM4000 B, Germany).

### Statistical Analyses

Data were shown by means ± standard error of the mean (S.E.M). The *p* values were calculated using two-tailed Student’s *t*-test or one-way analyses of variance (ANOVA) with Bonferroni *post hoc*. The GraphPad Prism Software was used for all analyses. Significance is reported as *p < 0.05, **p < 0.01, ***p < 0.001, and not significant values are not denoted except for emphasis.

## Results

### Systemic treatment With Bumetanide alleviated autistic-like behaviors in BTBR mice

**Figure 1:**
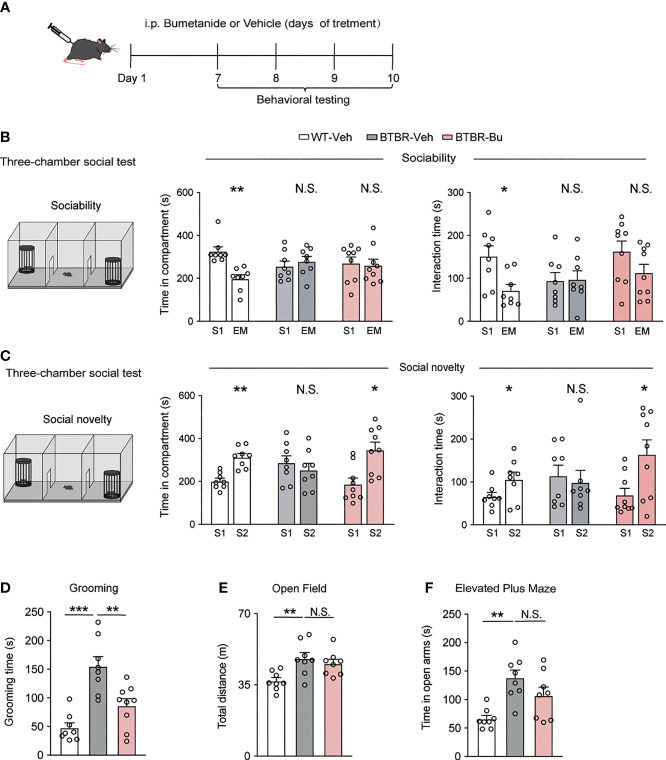
Systemic treatment with bumetanide alleviated autistic-like behavioral in BTBR mice. **(A)** Schematic representation of the experimental procedure. Male BTBR mice were injected intraperitoneally (i.p.) with bumetanide (10 mg/kg) or vehicle. **(B)** Time spent in the social stimulus or empty chamber and total interaction time with stimulus mouse or empty wire during sociability stage (WT-vehicle, n = 8; BTBR-vehicle, n = 8; BTBR-bumetanide, n = 9, paired Student’s *t*-test. Time in compartments, **p < 0.01, WT-vehicle S1 vs. EM; N.S., not significant, BTBR-vehicle S1 vs. EM; N.S., not significant, BTBR-bumetanide S1 vs. EM; interaction time, *p < 0.05, WT-vehicle S1 vs. EM; N.S., not significant, BTBR-vehicle S1 vs. EM; N.S., not significant, BTBR-bumetanide S1 vs. EM; S1 represent stranger stimuli mouse 1, EM represent empty cage). **(C)** Time spent in familiar or novel stimulus chamber and total interaction time with familiar or novel stimulus mouse during social novelty stage (WT-vehicle, n = 8; BTBR-vehicle, n = 8; BTBR-bumetanide, n = 9, paired Student’s *t*-test. Time in compartments, **p < 0.01, WT-vehicle S1 vs. S2; N.S., not significant, BTBR-vehicle S1 vs. S2; *p < 0.05, BTBR-bumetanide S1 vs. S2; interaction time, *p < 0.05 WT-vehicle S1 vs. S2; N.S., not significant, BTBR-vehicle S1 vs. S2; *p < 0.05, BTBR-bumetanide S1 vs. S2; S1 represent familiar stimuli mouse 1, S2 represent a novel stranger stimuli mouse 2). **(D)** Time spent self-grooming in grooming test (WT-vehicle, n = 8; BTBR-vehicle, n = 8; BTBR-bumetanide, n = 9. one-way ANOVA, F_(2,22)_ =16.77, *p* < 0.0001, Bonferroni *post hoc*: ***p<0.001, WT-vehicle vs. BTBR-vehicle; N.S., not significant, WT-vehicle vs. BTBR- bumetanide; **p<0.01; BTBR-vehicle vs. BTBR-bumetanide). **(E)** Total distance traveled in the open-field test (n = 8 each group; one-way ANOVA, F_(2,21)_ =6.453, *p* = 0.0065, Bonferroni *post hoc*: *p < 0.05, WT-vehicle vs. BTBR-vehicle; N.S., not significant; BTBR-vehicle vs. BTBR-bumetanide). **(F)** Time spent in the open arms elevated plus maze test (n = 8, each group. One-way ANOVA, F_(2,21)_ = 8.918, *p* = 0.0016, Bonferroni *post hoc*: ** *p* < 0.01, WT-vehicle vs. BTBR-vehicle; N.S., not significant; BTBR-vehicle vs. BTBR- bumetanide). All Data were presented as mean ± s.e.m.

BTBR mice were injected with bumetanide (10mg/kg, intraperitoneally, once daily) or vehicle followed by a series of behavioral tests (**Figure 1A**). To assess their social behavior, we used a three-chamber test to examine social approach (sociability stage) and social recognition (social novelty stage), as is shown in **Figures 1B, C**. In the sociability stage, compared to WT mice, BTBR mice spent less time both in the social stimulus chamber (stranger 1, S1) and direct interaction with the stimulus. Bumetanide administration failed to rescue the social ability of BTBR mice, which we assessed by counting the time in the social stimulus chamber and direct interaction time with the stimulus (**Figure 1B**). However, when presented with both familiar stimuli (S1) and novel stimulus (stranger 2, S2), BTBR mice spent more time exploring familiar stimulus than novel stimulus, while bumetanide-treated BTBR mice spent more time in the novel stimulus chamber (**Figure 1C**) and interacted more time with novel stimulus (**Figure 1C**) to the level of WT controls. Together, the increased social investigate time indicated that systemic treatment of bumetanide partially alleviated the impaired social phenotype in BTBR mice. Next, we examined repetitive behavior after systemic treatment with bumetanide in BTBR mice. The grooming test revealed that bumetanide-treated BTBR mice, compared to the untreated group, spent less time self-grooming (**Figure 1D**) which indicated that bumetanide improved the repetitive behaviors. Lastly, we tested basal locomotor activity and anxiety using open field and elevated plus maze, respectively. In the open field test, vehicle- or bumetanide-treated BTBR mice displayed similar total distance traveled, which was higher than WT mice as previously reported (**Figure 1E**) (33), implying a negligible impact of bumetanide on locomotor activity of BTBR mice. In the elevated plus maze, bumetanide-treated BTBR mice showed a non-significant change toward vehicle-BTBR levels (**Figure 1F**), suggesting that bumetanide did not alter anxiogenic effects (**Figure 1F**). Overall, treatment of bumetanide is efficacious to treat autistic-like behaviors and did not significantly affect the locomotor activity or anxiety behavior in BTBR mice.

### mPFC Region-Specific Infusion of Bumetanide Alleviated Social Deficits in BTBR Mice

**Figure 2:**
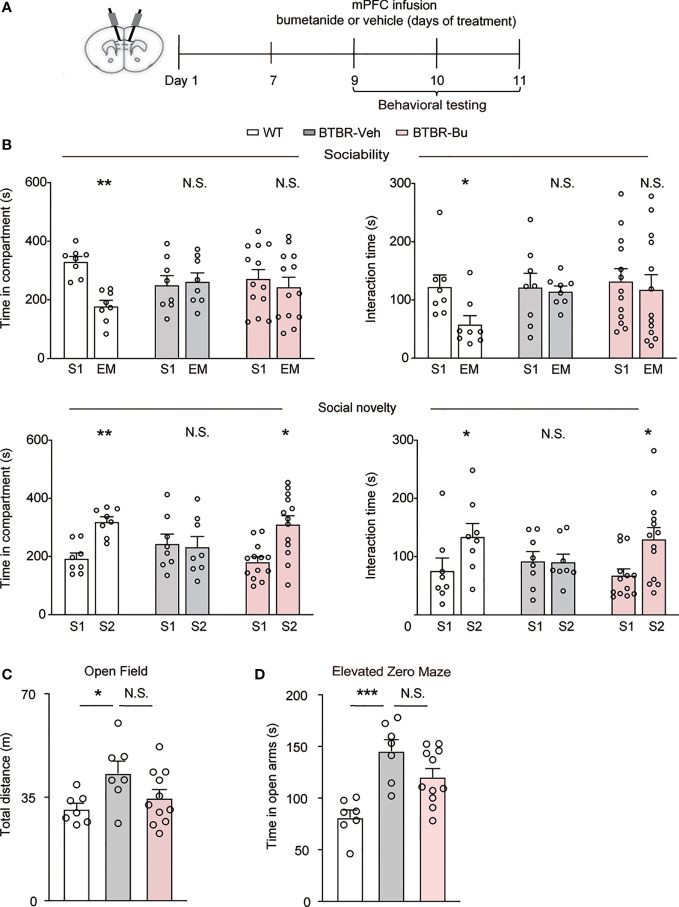
mPFC region-specific infusion with bumetanide alleviated social deficit in BTBR mice. **(A)** Schematic representation of the experimental procedure. Male BTBR mice were administrated with bumetanide (100 μM) or vehicle into mPFC. **(B)** Top: time spent in the social stimulus or empty chamber and total interaction time with stimulus mouse or empty wire during sociability stage (WT, n = 8; BTBR-vehicle, n = 8; BTBR-bumetanide, n = 13. paired Student’s *t*-test. Time in compartments, **p < 0.01, WT S1 vs. EM; N.S., not significant, BTBR-vehicle S1 vs. EM; N.S., not significant, BTBR-bumetanide S1 vs. EM; interaction time, *p < 0.05, WT S1 vs. EM; N.S., not significant, BTBR-vehicle S1 vs. EM; N.S., not significant, BTBR-bumetanide S1 vs. EM). Bottom: time spent in familiar or novel stimulus chamber and total interaction time with familiar or novel stimulus mouse during social novelty stage (paired Student’s *t*-test. Time in compartments, **p < 0.01, WT S1 vs. S2; N.S., not significant, BTBR-vehicle S1 vs. S2; *p < 0.05, BTBR-bumetanide S1 vs. S2; interaction time, *p < 0.05, WT S1 vs. S2; N.S., not significant, BTBR-vehicle S1 vs. S2; *p < 0.05, BTBR-bumetanide S1 vs. S2). **(C)** Total distance traveled in the open field test (WT, n = 7; BTBR-vehicle, n = 7; BT BR-bumetanide, n = 11, one-way ANOVA, F_(2,22)_ = 3.651, *p* = 0.0427, Bonferroni *post hoc*: *p < 0.05, WT vs. BTBR-vehicle; N.S., not significant, BTBR-vehicle vs. BTBR-bumetanide). **(D)** Time spent in the open arm during 5 min in elevated zero maze test (WT, n = 7; BTBR-vehicle, n = 7; BTBR-bumetanide, n = 11, one-way ANOVA, F_(2,22)_ = 11.74, *p*=0.0003, Bonferroni *post hoc*: ***p<0.001, WT vs. BTBR-vehicle; N.S., not significant, BTBR-vehicle vs. BTBR-bumetanide). Data were presented as mean ± s.e.m.

The mPFC has been recognized as a critical region in the regulation of social behavior. To examine whether mPFC region-specific delivery of bumetanide could affect social behaviors in BTBR mice, we employed region infusion with bumetanide (100 μM, 0.5 ul each side) to the mPFC in BTBR mice (**Figure 2A**). In the sociability stage of three-chamber test, similarly to systemic treatment, intra-mPFC bumetanide treatment had no significant effect in improving sociability of BTBR mice (**Figure 2B**, Top), as measured by both time spent in the chamber and direct interaction time with social stimulus. In social novelty stage, intra mPFC bumetanide ameliorates the social novelty preference of BTBR mice, reflected both by time investigated in the novel social stimulus (S2) chamber and direct interaction time with the novel stimulus as compared to the familiar stimulus (S1) (**Figure 2B**, Bottom). This suggested that bumetanide alleviated social deficit in a mPFC region-specific way. Furthermore, in the open field test, no significant differences were observed with infusion of bumetanide to mPFC in total distance of locomotion behavior compared to vehicle-treated BTBR mice (**Figure 2C**). In the elevated zero maze test, vehicle-BTBR mice spent more time in the open arm than WT mice, and bumetanide-treated BTBR mice spent similar amounts of time in the open arm as vehicle group suggesting that bumetanide did not alter anxiogenic effects (**Figure 2D**). These data suggest that the effectiveness of bumetanide in alleviating social deficit is a mPFC region-specific way without causing nonspecific behavioral effects.

### Formation and Brain Distribution of the Nanoparticles Following Intra-mPFC Administration

**Figure 3:**
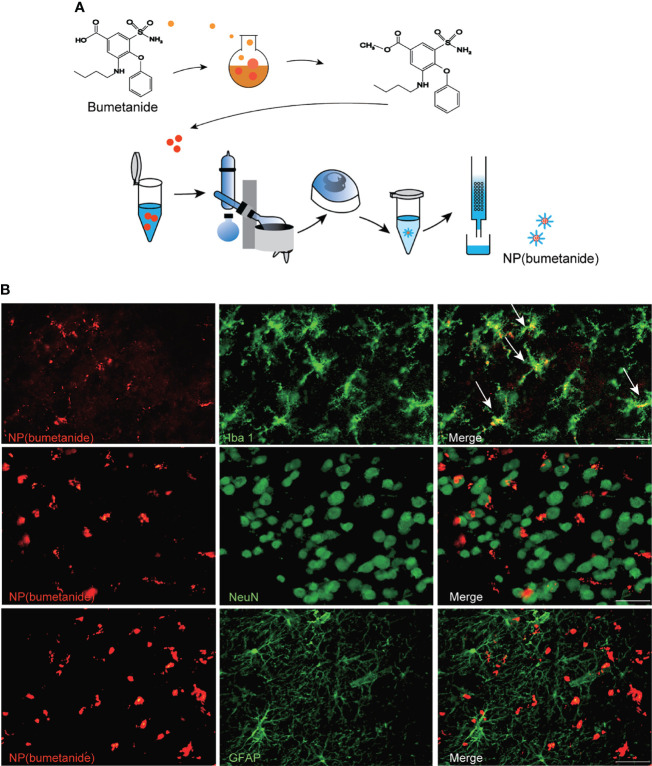
NP(bumetanide) selectively targeting microglia of mPFC *in vivo*. **(A)** Schematic representation of the nanoformulated bumetanide experimental procedure. **(B)** Representative images of the fluorescence staining. NP(bumetanide) (Rhodamine-label, Red), microglia (Iba1, Green), Neuron (NeuN, Green), and astrocyte (GFAP, Green) and merged image in mPFC slices of BTBR mice, and co-localization of NP (bumetanide) and Iba1 are shown as arrow. Scale bar 100 μm.

We wrapped bumetanide in a core of PEG-PLA as illustrated in **Figure 3A**. To explore the cell distribution of the NP(bumetanide) in BTBR mice, we performed rhodamine-labeled nanoparticles to directly visualize NP(bumetanide). We then administered rhodamine-labeled NP(bumetanide) (100 μM) into mPFC and sacrificed the mice. Immunostaining of several markers for different types of cells to determine the co-localization of NP(bumetanide). As shown in **Figure 3B**, we examined Iba1, which is a typical microglia marker, and found that NP(bumetanide) accumulated in microglia. We further examined NeuN and GFAP, which represent neurons and astrocytes, respectively, and found no overlap of NeuN-positive cells and astrocytic cells with NP(bumetanide). These findings suggest that NP(bumetanide) targets microglia *in vivo*.

### NP (bumetanide) Infusion in the mPFC Alleviated Social Deficits in BTBR Mice

**Figure 4:**
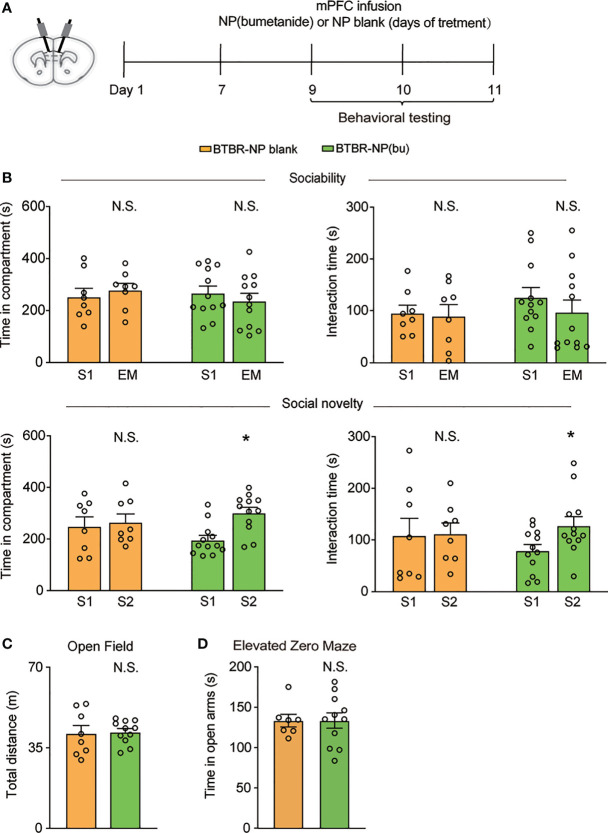
mPFC region specific administration of NP(bumetanide) alleviated social deficits in BTBR mice. **(A)** Schematic representation of the experimental procedure. Male BTBR mice were intra-mPFC infusion of NP(bumetanide) (100 μM) or NP blank. **(B)** Top: time spent in the social stimulus or empty chamber and total interaction time with stimulus mouse or empty wire during sociability stage (BTBR-NP blank, n = 8; BTBR-NP(bumetanide), n = 12, paired Student’s *t*-test. Time in compartments, N.S., not significant, BTBR-NP blank S1 vs. EM; N.S., not significant, BTBR-NP(bumetanide) S1 vs. EM; interaction time, N.S., not significant, BTBR-NP blank S1 vs. EM; N.S., not significant, BTBR-NP(bumetanide) S1 vs. EM). Bottom: time spent in familiar or novel stimulus chamber and total interaction time with familiar or novel stimulus mouse during social novelty stage (paired Student’s *t*-test. Time in compartments, N.S., not significant, BTBR-NP blank S1 vs. S2; *p<0.05, BTBR-NP(bumetanide) S1 vs. S2; interaction time, N.S., not significant, BTBR-NP blank S1 vs. S2; *p<0.05, BTBR-NP(bumetanide) S1 vs. S2). **(C)** Total distance traveled in the open-field test (BTBR-NP blank, n = 8; BTBR-NP(bumetanide), n = 11, unpaired student *t*-test, N.S., not significant). **(D)** Time spent in the open arm in elevated zero maze test (BTBR-NP blank, n = 7; BTBR-NP(bumetanide), n = 11; unpaired student *t*-test, N.S., not significant). All data were presented as mean ± s.e.m.

Next, we further examined the therapeutic effect of NP(bumetanide) in BTBR mice. Behavior tests were performed in BTBR mice after intra-mPFC NP(bumetanide) (100 μM) infusion (**Figure 4A**). Like NP-blank-treated BTBR mice, sociability stage analysis revealed no significant change in the amount of time spent in the S1 chamber or direct with S1 (**Figure 4B**, Top) in NP(bumetanide)-treated BTBR mice. We detected a statistically significant difference for the social novelty stage, showing that NP(bumetanide)-treated BTBR mice have increased social approach in this social novelty stage, while measured by time spent in the novel chamber and interaction time with the novel stimulus (**Figure 4B**, bottom). In addition, no changes were observed in total distance traveled between groups in the open field test (**Figure 4C**). NP(bumetanide)- and NP-blank-treated-BTBR mice spent nearly equal time in the open arm in the elevated zero-maze test, suggesting that NP(bumetanide) treated mice did not have increased anxiety levels (**Figure 4D**). Taken together, these data suggest that NP(bumetanide) is effective in alleviating social deficits in a non-neuronal manner without causing non-specific behavioral effects.

### NP (bumetanide) Alleviated Social Deficits in BTBR Mice Necessitate Microglia in mPFC

**Figure 5:**
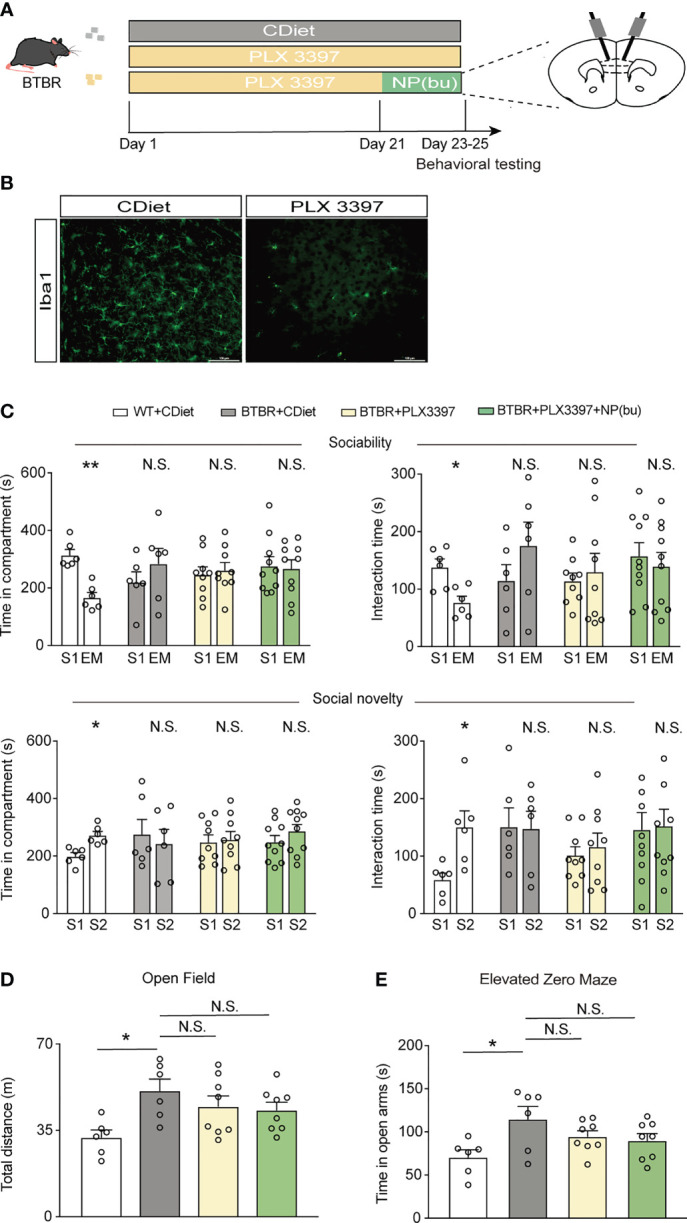
NP(bumetanide) improved social behaviors in BTBR mice necessitate microglia in mPFC. **(A)** Schematic representation of the experimental procedure. Male BTBR mice were pretreated diets formulated with PLX3397 (CSF1R antagonist) or control diets for 25 days. NP(bumetanide) was provided for 3 times from day 21 and behavior tests were tested. **(B)** Representative Iba1 immunofluorescent staining from the mPFC region of CDiet (Left) and PLX3397 (Right)- treated BTBR mice. Scale bar 100 μm. **(C)** Top: time spent in social stimulus or empty chamber and total interaction time with stimulus mouse or empty wire during sociability stage (WT + CDiet, n = 6; BTBR + CDiet, n = 6; BTBR + PLX3397, n = 9, BTBR + PLX3397 + NP(bumetanide), n = 10, paired Student’s *t*-test. Time in compartments, **p<0.01, WT + CDiet S1 vs. EM; N.S., not significant, BTBR + CDiet S1 vs. S2; N.S., not significant, BTBR+PLX3397 S1 vs. S2; N.S., not significant, BTBR+PLX3397+NP(bumetanide) S1 vs. EM; interaction time, *p<0.05, WT + CDiet S1 vs. EM; N.S., not significant, BTBR + CDiet S1 vs. EM; N.S., not significant, BTBR+PLX3397 S1 vs. EM; N.S., not significant, BTBR+PLX3397+NP(bumetanide). Bottom: time spent in familiar or novel stimulus chamber and total interaction time with familiar or novel stimulus mouse during social novelty stage (paired Student’s *t*-test. Time in compartments, *p<0.05, WT + CDiet S1 vs. S2; N.S., not significant, BTBR + CDiet S1 vs. S2; N.S., not significant, BTBR + PLX3397 S1 vs. S2; N.S., not significant, BTBR + PLX3397 + NP(bumetanide) S1 vs. S2; interaction time, *p<0.05, WT + CDiet S1 vs. S2; N.S., not significant, BTBR + CDiet S1 vs. S2; N.S., not significant, BTBR + PLX3397 S1 vs. S2; N.S., not significant, BTBR + PLX3397 + NP(bumetanide) S1 vs. S2). **(D)** Total distance traveled in the open-field test (WT + CDiet, n = 6; BTBR + CDiet, n= 6; BTBR + PLX3397, n =8; BTBR + PLX3397 + NP(bumetanide), n = 8, one-way ANOVA, F(3,24) = 3.704, *p*=0.0254, Bonferroni *post hoc*: *p < 0.05, WT + CDiet vs. BTBR + CDiet; N.S., not significant, BTBR + CDiet vs. BTBR + PLX3397; N.S., not significant, BTBR + CDiet vs. BTBR + PLX3397 + NP(bumetanide). **(E)** Time spent in the open arm in elevated zero maze test (WT + CDiet, n = 6; BTBR + CDiet, n= 6; BTBR + PLX3397, n =8; BTBR + PLX3397 + NP(bumetanide), n = 8, one-way ANOVA, F(3,24) = 3.326, *p*=0.0366, Bonferroni *post hoc*: *p < 0.05, WT + CDiet vs. BTBR + CDiet; N.S., not significant, BTBR + CDiet vs. BTBR + PLX3397; N.S., not significant, BTBR + CDiet vs. BTBR + PLX3397 + NP(bumetanide). Data were presented as mean ± s.e.m.

To explore whether the therapeutic efficacy of NP(bumetanide) was due to the cell-specific mechanism, we pretreated BTBR mice with PLX3397 to deplete microglia. PLX3397 is an orally selective CSF1R kinase inhibitor that has been reported to pharmacologically deplete more than 99% of microglia in the brain (34). Five-week-old BTBR mice were pre-treated with PLX3397 (290 mg/kg, standard chow) for 25 days with age-matched controls on standard chow, and infusion with NP(bumetanide) or vehicle in the mPFC for three times from day 21. Mice were tested on behavior tests (**Figure 5A**). Immunostaining for Iba1 showed a reduction in microglia in the mPFC of BTBR mice (**Figure 5B**). In the sociability stage, microglia-depleted BTBR mice spent less time on exploration of social stimulation chamber and social stimulus similar to the BTBR mice, indicating that microglia-depleted BTBR mice had no improvement in social approach in this stage (**Figure 5C** Top). In contrast, NP(bumetanide)-treated microglia depletion BTBR mice also spent less time in the social stimulation chamber compared to WT control. Next, we found that microglia depletion BTBR mice spent less time on exploration of S2 than on S1 in the social novelty stage, showing that microglial depletion throughout the brain had no improvement on social deficits in BTBR mice. In particular, NP(bumetanide)-treated microglia depletion BTBR mice showed no significant improvement in investigating of S2 over S1 (**Figure 5C** Bottom), showing that microglia depletion blocked the therapeutic effect of NP (bumetanide) in BTBR mice. In addition, no changes were observed in total distance traveled between BTBR groups in the open field test (**Figure 5D**). Neither depletion of microglia group nor NP(bumetanide)-treated microglia depletion BTBR group have increased anxiety levels in BTBR mice compared to BTBR control group (**Figure 5E**). Together, these behavioral results demonstrate that microglia depletion alone has no effects on social behaviors, and NP(bumetanide) improved social behaviors necessitates the presence of mPFC microglia in BTBR mice.

## Discussion

**Figure 6:**
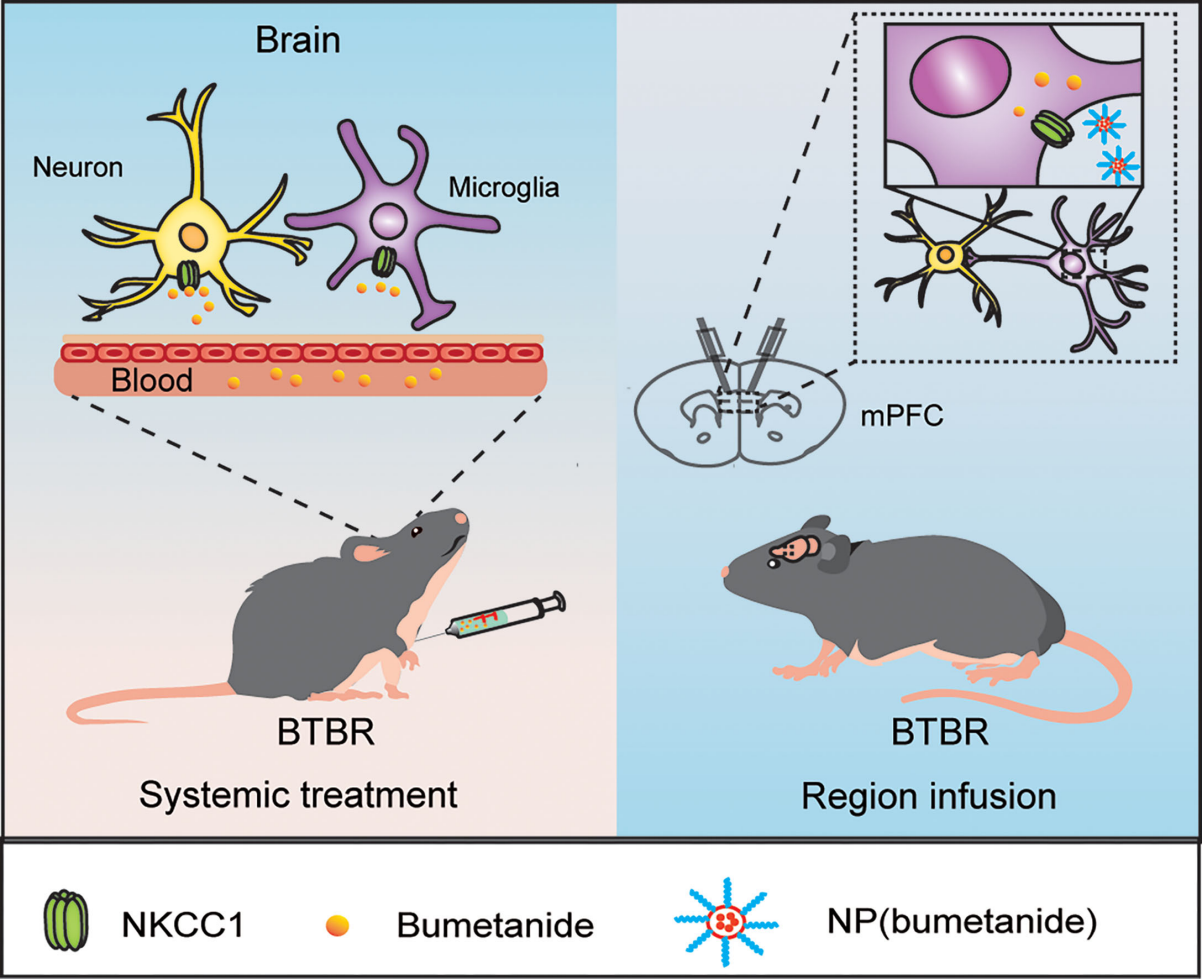
A proposed work model by bumetanide and NP(bumetanide) in the BTBR mice.

In this study, we reported that bumetanide alleviated social deficits in BTBR mice in a region- and cell type-specific manner **(** **Figure 6****)**. Specifically, both systemic and mPFC region-specific delivery of bumetanide treated social deficits without influencing basic locomotor activity and anxiety-like behaviors in BTBR mice. Moreover, using a nanoparticulate drug delivery system, we showed that NP(bumetanide) also has a therapeutic effect in BTBR mice, in a microglia-dependent fashion. Mechanistically, NP(bumetanide) selectively targets microglia but not neurons or astrocytes and it was enough to alleviate social novelty impairments in BTBR mice in a mPFC region-specific way. These findings demonstrated a novel therapeutic strategy for ASD and a new insight into the function of bumetanide on immune cells.

Although preclinical and clinical studies have reported bumetanide attenuates symptoms of ASD (7, 9, 14, 35), two phase III clinical trials of bumetanide in ASD patients were terminated because of ineffective outcomes. These failure outcomes may be due to the difference in methodology including the dosage of bumetanide, multi-center trials, the possible high dropout rate, and the variability population with ASD (36). The precise effectiveness and action of bumetanide should be clarified. The effects of bumetanide were identified previously in maternal treatment of ASD mice (FRX and VPA) offspring (7). In our study, the results are consistent with previous findings in the systemic treatment of adolescent mice. The partial alleviation in social deficit alterations (improvement in social novelty exploration) that we observed in BTBR mice is implicated with the efficiency of bumetanide in treating social deficits, which is directly relevant to a core symptom of ASD. Despite the differences in the experimental methods, including model differences, inconsistent age of treatment starting, and treatment duration, our present study demonstrated reproducibility of bumetanide treatment in multiple models of ASD.

Furthermore, our results showed that bumetanide reduced repetitive behavior in BTBR mice. Indeed, BTBR mice are not the only ones that responded to bumetanide in several ASD mice models, including FRX and VPA, which also have improved repetitive behavior outcomes after treatment with bumetanide (7, 8, 37). Our results not only confirm previous findings as they provide evidence for the therapeutic effect of bumetanide, and complement in immune alterations found in rodent models of ASD.

Based on the wide tissue and cellular expression of NKCC1, combined with the low brain penetration and non-selective effects of bumetanide, it is proper to question if the effects of bumetanide in ASD are peripherally or centrally mediated. Recent investigations validated the role of NKCC1 as a pharmacological effector in rodent models of neurological diseases (38). Indeed, bumetanide modulated neuronal electrical parameters *in vitro* brain slice (7, 39), suggesting a central actions of bumetanide in ASD models. Enhancement of inhibitory neurotransmission by treatment with low doses of benzodiazepines improved deficits in autistic behavior in BTBR mice (40). To elucidate the roles of bumetanide at central nervous system level in ASD models, we investigated the effect of region-specific delivery of bumetanide in BTBR. The mPFC processes information related to social behavior especially in exploration of novelty social target (17), and the dysfunction of E/I ration in the mPFC contribute to ASD-like behavior in mice (15). We demonstrate the effects of mPFC-specific delivery of bumetanide and found this pattern was sufficient to improve social novelty exploration, similar to the therapeutic effects of the systemic way, suggesting a centrally-mediated mechanism by which bumetanide exerts its therapeutic effect on mice model of ASD. This finding is also consistent with the evidence that restoring the E/I balance is sufficient to ameliorate social approach in the VPA rodent models of ASD, as reported in previous findings (41), which could be due to manipulated E/I balance in the mPFC. Therefore, these findings emphasize the importance of the mPFC for the effect of bumetanide, and that the effects of systemic bumetanide treatment may be dependent on central targets, namely the mPFC region in the BTBR.

Besides the neuronal actions of bumetanide and its precise cellular targets of bumetanide in ASD, the effects of bumetanide on diverse cell types should also be investigated. Importantly, the pathophysiological factors of ASD include immune mechanism, and treatment targeting immune factors may be considered a novel therapeutic strategy for ASD. Microglia are the resident immune cells of the brain, and previous studies have shown microglia dysfunction in both patients with ASD and in preclinical models (26, 42, 43). We observed that NP(bumetanide) accumulated in microglia, and the infusion of NP(bumetanide) in the mPFC improved social novelty in BTBR strain. In order to provide mechanistic evidence that microglia-specific targeting can improve social deficit in BTBR mice, we established a pharmacological depletion of microglia in BTBR mice using a CSF1R inhibitor. Microglia are the only resident immune cells of the brain that express CSF1R, and it has been identified that short-term administration (3 weeks) of PLX3397 can eliminate virtually all microglia in the brain (34). Interestingly, in this study, no improvement was observed in microglia depletion BTBR mice as determined by social tests. It seems that microglia function is subtle in the brain and plays a complex role of microglia in ASD brain, as elimination of microglia alone is not sufficient to rescue social behavior. Microglia could secrete soluble factors and directly interact with neurons, participating in brain function. Indeed, depletion of microglia at an early development stage has led to autistic-like behavior in mice, which was associated with the deficit of synaptic pruning (44). Modulation of microglial function, rather than eliminating it, seems to be a more efficient strategy in ASD, and future research could use genetic manipulation tools to target microglia to further answer this question.

Crucially, the depletion of microglia significantly blocked the beneficial effects of NP(bumetanide) treatment in BTBR mice. Further understanding of the underlying molecular mechanisms of how bumetanide regulates microglia function remains to be elucidated. NKCC1 is expressed in glial cells, and a single-cell transcriptomic study (BRAIN-SAT database) has supported NKCC1 expression in microglia (21), which consisted of a recent study showing microglial NKCC1 expression in the brain (45), but its role in neurodevelopment conditions like ASD remains unknown. Researchers found that bumetanide attenuated LPS-induced acute lung injury by inhibiting NKCC1-mediated macrophage volume alteration and inflammatory function in a mouse model of LPS-induced lung injury (30). This indicates that the beneficial actions of NP(bumetanide) may be based on the anti-inflammatory action caused by the inhibition of NKCC1 in the microglia. However, there is an intriguing finding that the direct administration of bumetanide into the brain *in vivo* displayed the opposite effect of a systemic administration and can be explained by changes in K^+^ efflux mechanism in microglia (45). Therefore, the precise mechanism through the NKCC1 inhibitor bumetanide modulates the function of microglia in BTBR mice needs to be further investigated. We reasoned about several underlying mechanisms as follows: firstly, systemic treatment of bumetanide recently was reported to reduce inflammatory response in the brain of LPS-induced mice (45), and in the lung of LPS-induced lung injury mice (30), suggesting the therapeutic effects of systemic treatment of bumetanide in BTBR mice could be partially due to anti-inflammatory action. However, due to the non-selective action of bumetanide, neuronal and non-neuronal (both periphery and central) cells may be involved in this action; secondly, In this study, we used a nanoparticle system to deliver bumetanide, and the NP(bumetanide) would be easier to induce the phagocytosis function of microglia given the microglia are detector cells in the brain. Microglia phagocytosis is often reported to be suppressed in ASD models due to the lack of elimination of synapses by microglia. For instance, *Fmr1* KO mice (Fragile X syndrome) showed impaired microglia-mediated synaptic elimination during the developmental period in CA region of the brain (46). TREM2 (a risk gene of Alzheimer’s disease related to microglial phagocytosis) KO mice displayed autistic-like behaviors and reduced microglial synaptic elimination (47). More recently reported that enhanced protein synthesis in microglia increased the number of excitatory synapses in the mPFC and caused autistic-like behavior in mice which may be related to a decrease in microglia motility and phagocytosis of synapses (48). In a maternal immune activation (MIA) mice model of ASD, minocycline treatment suppressed the inflammatory activation of microglia and restored the phagocytic of microglia whilst alleviating the autistic-like behaviors (49). Restoration of the motility and phagocytosis of microglia may be involved in NP(bumetanide)’s action in BTBR mice; thirdly, although bumetanide is a well-known inhibitor of NKCC1, it is possible to find out that the effects of bumetanide are due to other pharmacological effects independent of the NKCC1 inhibition.

Finding the specific cellular mechanism of bumetanide’s modulation of behavioral and cellular features related to ASD can provide instrumental information to better understand the pathophysiology of ASD. Using the nanoparticle drug system as a tool, we also demonstrated that NP(bumetanide) alleviates social behavior in an mPFC-microglia-dependent way in BTBR mice. The proposed link between bumetanide and microglia, which is involved in the recovery of social deficit in BTBR mice, provides an important set of evidence relating to neuroimmunology and ASD, as well as generating scope for future therapeutic targets.

## Data Availability Statement

The raw data supporting the conclusions of this article will be made available by the authors, without undue reservation.

## Ethics Statement

The animal study was reviewed and approved by Animal Ethics Committee of East China Normal University.

## Author Contributions

FL and XLG conceptualized the study; HL, XG, XS, TLZ, BM, TTZ, and H-TZ performed animal research and data analysis; HL, VB-J, W-GL, and FL wrote the manuscript with contributions from all authors. All authors read and approved the final manuscript.

## Funding

This study was supported by grants from the National Natural Science Foundation of China (82125032, 81930095 and 81761128035), the Science and Technology Commission of Shanghai Municipality (19410713500 and 2018SHZDZX01), the Shanghai Municipal Commission of Health and Family Planning (GWV-10.1-XK07, 2020CXJQ01, 2018YJRC03), the Shanghai Clinical Key Subject Construction Project (shslczdzk02902), the Guangdong Key Project (2018B030335001), and innovative research team of high-level local universities in Shanghai.

## Conflict of Interest

The authors declare that the research was conducted in the absence of any commercial or financial relationships that could be construed as a potential conflict of interest.

## Publisher’s Note

All claims expressed in this article are solely those of the authors and do not necessarily represent those of their affiliated organizations, or those of the publisher, the editors and the reviewers. Any product that may be evaluated in this article, or claim that may be made by its manufacturer, is not guaranteed or endorsed by the publisher.
